# Gamification is not just fun: the effect of gamification design elements on shaping intrinsic motivation and learning persistence

**DOI:** 10.1186/s40359-026-05046-8

**Published:** 2026-07-02

**Authors:** Chi-Hung Lo

**Affiliations:** Department of Industrial Design, Tunghai University, Taichung, Taiwan

**Keywords:** Game-based learning, Gamification design elements, Intrinsic motivation, Learning Persistence

## Abstract

As digital learning becomes more common in higher education, learners in online and blended learning environments may encounter challenges such as insufficient engagement, distraction, and course non-completion. Therefore, promoting sustained participation and long-term learning persistence has become an important issue in educational technology and instructional design. In recent years, gamification has attracted increasing attention because of its potential to support learner engagement, feedback, progress visualization, and social interaction. However, its longer-term motivational mechanisms in educational contexts remain insufficiently clarified. This study examined the relationships among gamification design elements, intrinsic motivation, and learning persistence in a 16-week blended digital game-based learning course. A one-group post-intervention survey design was adopted. A total of 337 students from a university in Taichung, Taiwan participated in the study, and data were collected through paper-based self-report questionnaires at the end of the course. Exploratory factor analysis and reliability analysis indicated acceptable construct validity and internal consistency of the measurement scales. Structural equation modeling showed that gamification design elements were significantly and positively associated with intrinsic motivation, and intrinsic motivation was significantly and positively associated with learning persistence. Gamification design elements were also directly associated with learning persistence. The mediation analysis further indicated that intrinsic motivation partially mediated the relationship between gamification design elements and learning persistence. The model fit indices suggested acceptable model fit, and the standardized path coefficients indicated meaningful but moderate positive associations. Overall, the findings provide preliminary empirical support for the proposed model and suggest that need-supportive gamified instructional design may be related to higher intrinsic motivation and stronger learning persistence. The study contributes to gamified learning research by interpreting gamification design as a need-supportive instructional structure rather than merely as a reward-based strategy.

**Supplementary Information:**

The online version contains supplementary material available at https://doi.org/10.1186/s40359-026-05046-8.

## Introduction

Online learning platforms, learning management systems, and blended learning models have gradually become standard practices in higher education and vocational education. Compared with traditional face-to-face classrooms, digital learning provides greater flexibility and accessibility and enables the documentation of learning processes for learning analytics and instructional decision-making [55]. However, increased access to digital resources does not automatically lead to effective learning outcomes. In digital learning environments, learners are often required to regulate their own goals, time, strategies, motivation, and learning progress. Students with insufficient self-regulated learning skills may experience difficulties in sustaining attention, managing learning tasks, monitoring comprehension, and transforming digital resources into meaningful learning outcomes [8, 56]. Empirical studies have also shown that online and digital learning environments may weaken students’ behavioral and emotional engagement when interaction, feedback, and instructional presence are insufficient [7, 20]. In addition, because digital learning often occurs through internet-connected devices, learners may be more vulnerable to off-task behaviors, multitasking, and digital distraction, which can reduce attention and interfere with learning performance [1, 27]. Course withdrawal and non-completion have also been widely reported in online learning, especially in MOOCs and self-paced courses, where low motivation, limited interaction, poor time management, and weak self-regulated learning are closely associated with dropout or non-completion [21, 52]. Therefore, the major challenge of digital learning is not only access to content, but also how to sustain learners’ motivation, engagement, and persistence throughout the learning process.

Among different instructional implementation strategies, gamification has gained popularity in both educational practice and academic research. This is because of its actionable design language and scalable applications. Following Deterding et al. [13], this study defines gamification as the use of game design elements in non-game contexts. In the present educational context, gamification is understood as an instructional design strategy that integrates selected game elements into learning activities without replacing the original learning objectives. This interpretation is also consistent with Kapp [23] view that gamification uses game-based mechanics, aesthetics, and game thinking to engage learners, motivate action, promote learning, and solve instructional problems. Accordingly, the gamification design in this study includes points, badges, leaderboards, levels, quests, and Intrinsic motivation to experience stimulations, which are used to visualize learning progress, provide feedback, strengthen goal orientation, and encourage sustained participation. Gamified instructional design can support learning motivation and engagement by incorporating mechanisms such as immediate feedback, progress visualization, narrative contexts, and social interaction. Immediate feedback helps learners recognize the consequences of their actions and adjust their learning strategies in a timely manner, while progress visualization, such as points, badges, levels, or progress bars, makes learning achievement and task completion more visible [38, 42]. Narrative contexts can provide meaningful scenarios and game fiction that connect learning tasks with goals, roles, and Intrinsic motivation to experience stimulations, thereby increasing learners’ sense of immersion and task relevance [9, 48]. Social interaction, including cooperation, competition, peer comparison, and collaborative problem-solving, can further strengthen learner participation and behavioral engagement in gamified learning environments [31, 48]. Therefore, in the present study, gamification is not treated simply as the addition of rewards, but as a structured instructional design approach that combines feedback, visible progress, meaningful context, and social participation to support students’ sustained engagement. Unlike games designed for entertainment, gamification focuses on improving the interactivity, feedback quality, and sense of goal orientation in the learning processes without modifying the learning. For educators, gamification provides concrete instructional design tools that can make learners’ effort, progress, and achievement more visible. Prior studies suggest that feedback, progress visualization, and appropriately structured challenges can help learners recognize task outcomes, monitor their learning progress, and adjust their learning strategies in a timely manner [38, 42]. These mechanisms may support sustained participation by reducing uncertainty during task completion and by helping learners perceive clearer goals, progress, and achievement during the learning process. Nevertheless, the effectiveness of gamification in educational contexts is not standardized. Although gamification has been widely used to increase students’ motivation and participation, prior meta-analyses, systematic reviews, and longitudinal studies have raised concerns about whether its effects can be sustained over time and whether increased participation necessarily leads to persistent learning. For example, Sailer and Homner’s [48] meta-analysis showed that gamification had significant but relatively small effects on cognitive, motivational, and behavioral learning outcomes, suggesting that its effectiveness may depend on specific design conditions rather than the mere use of game elements. Similarly, Li et al. [30] reported mixed findings regarding the effects of gamification on learning outcomes, indicating that higher engagement does not always translate into stronger academic performance. From a longitudinal perspective, Rodriguez et al. [46] found that the effects of gamification may decline after the initial implementation period because of the novelty effect, raising questions about the long-term stability of gamified learning interventions. In addition, Kim et al. [24] suggested that gamified interventions may be more effective in initiating short-term behavioral change than in maintaining long-term behavioral outcomes. These findings indicate an important research gap: while gamification may increase students’ immediate participation and engagement, more empirical evidence is needed to examine whether such participation can develop into sustained learning motivation, persistent learning behavior, and stable learning outcomes. Previous research has reported both the potential benefits and limitations of gamification in educational contexts. On the one hand, meta-analytic and review studies have shown that gamification can enhance students’ motivation, engagement, participation, and learning outcomes when game elements are meaningfully aligned with instructional goals [30, 45, 48]. For example, gamified elements such as points, badges, leaderboards, and progress indicators may encourage learners to participate more actively, complete learning tasks, and maintain short-term behavioral engagement. On the other hand, researchers have also cautioned that gamification should not rely excessively on external rewards. Ratinho and Martins [45] noted that points, badges, and rankings are commonly used to motivate students, but motivation may decline over time if gamification mainly depends on reward-driven mechanisms. Similarly, Almeida et al. [2] found that badges, leaderboards, competitions, and points are among the game design elements most frequently associated with negative effects, including motivational problems and lack of effect. Mogavi et al. [40] further showed that learners may become overly focused on gamified rewards or competition, which can distract them from meaningful learning. Therefore, this study positions gamification not merely as a reward system, but as an instructional design approach that should balance extrinsic incentives with meaningful learning tasks, feedback, autonomy, and sustained engagement. Self-determination theory and related gamification research suggest that overreliance on extrinsic rewards may shift learners’ attention from the intrinsic value of learning to the external outcomes attached to learning activities. Deci, Koestner, and Ryan’s [11] meta-analysis showed that expected tangible rewards may undermine intrinsic motivation, particularly when learners perceive such rewards as controlling rather than autonomy-supportive. In gamified learning contexts, this concern is especially relevant because commonly used elements such as points, badges, rankings, and leaderboards may encourage learners to focus on reward accumulation or social comparison rather than meaningful learning. Hanux and Fox [18] found that students in a gamified course showed lower motivation and satisfaction over time, and that reduced intrinsic motivation negatively affected final exam performance. Similarly, Mogavi et al. [40] reported that gamification misuse may occur when learners become overly fixated on badges, points, streaks, or competition, which can distract them from actual learning tasks and weaken learning performance. Therefore, this study argues that gamified instructional design should avoid treating extrinsic rewards as the primary source of motivation and should instead combine rewards with autonomy-supportive tasks, meaningful feedback, and opportunities for intrinsic engagement. These mixed findings suggest that the effects of gamification depend heavily on design choices, learning context, and the way game elements are combined with instructional goals. For example, reward-based elements such as points, badges, and leaderboards may increase participation, but they may also encourage reward accumulation or social comparison when they are perceived as controlling rather than autonomy-supportive [11, 18, 40]. By contrast, feedback, progress visualization, meaningful challenges, narrative contexts, and social interaction may support learners’ sense of competence, autonomy, and relatedness when they are aligned with learning tasks and instructional objectives [31, 38, 48]. Therefore, the present study does not examine the independent causal effect of each individual game element. Instead, it examines how an integrated gamified instructional design is perceived by learners as autonomy support, competence support, and relatedness support.

Intrinsic motivation and enabling persistent learning are often treated as closely related concepts. However, they differ both in theoretical meaning and in terms of behavioral manifestation. Intrinsic motivation emphasizes engagement resulting from interest, Intrinsic motivation to know, and personal growth. However, persistent learning focuses on whether learners are willing to maintain engagement, delay withdrawal, and complete tasks over time. This is especially the case when difficulties arise. Learners may display high initial participation due to originality effects but are unable to sustain engagement in later stages. They may also complete tasks under external pressure without any increase in intrinsic motivation. These observations imply that understanding how gamification improves learning outcomes requires more than examining short-term participation. More critically, it is necessary to clarify whether gamification design elements can enhance learners’ intrinsic motivation. This, on the other hand, turns motivation into persistent learning.

A review of the existing literature reveals several gaps. Although the relationship between gamification and intrinsic motivation has been widely examined, several issues remain unresolved. First, prior studies have often treated gamification either as a general instructional intervention or as a set of visible game elements, such as points, badges, leaderboards, challenges, and feedback. However, less attention has been paid to how an integrated gamified instructional design is perceived by learners as psychological need support. From the perspective of self-determination theory, gamification may influence learning by supporting learners’ needs for autonomy, competence, and relatedness, thereby strengthening intrinsic motivation [31, 57]. Therefore, the present study does not aim to identify the independent causal effect of each individual game element. Instead, it examines whether students’ overall perceptions of gamified instructional design can be organized as autonomy support, competence support, and relatedness support. Second, previous studies have often examined intrinsic motivation as a general outcome variable, while fewer studies have considered its multidimensional structure in gamified learning contexts. This limits our understanding of whether gamified instructional design supports different forms of intrinsic motivation, such as intrinsic motivation to know, intrinsic motivation toward accomplishment, and intrinsic motivation to experience stimulation. Third, although some studies have examined mediating processes linking gamification to learning outcomes, such as engagement, satisfaction, intrinsic motivation, and learning regulation [43, 59], the relationship between need-supportive gamified design, multidimensional intrinsic motivation, and learning persistence still requires further empirical clarification. In particular, many studies have emphasized short-term engagement, participation, or immediate performance, while less attention has been paid to whether gamified instructional design can support sustained learning involvement over a longer instructional period. Therefore, the present study addresses these limitations by examining gamification design elements as an integrated need-supportive instructional design, adopting Vallerand et al.’s tripartite classification of intrinsic motivation, and testing whether these motivational processes are associated with learning persistence in a 16-week blended digital game-based learning course.

Although the relationship between gamification and intrinsic motivation has been widely examined in previous studies, several issues remain unresolved. First, many prior studies have treated gamification as a general instructional intervention or compared gamified and non-gamified learning environments, rather than clarifying how specific gamification design elements function as psychological need-supportive mechanisms. As a result, it remains unclear how game elements such as feedback, progress visualization, challenges, achievement indicators, and social interaction are translated into autonomy support, competence support, and relatedness support. Second, previous studies have often examined intrinsic motivation as a general outcome variable, but fewer studies have considered its multidimensional structure. This limits our understanding of whether gamified learning supports different forms of intrinsic motivation, such as intrinsic motivation to know, intrinsic motivation toward accomplishment, and intrinsic motivation to experience stimulation. Third, many studies have emphasized short-term engagement, participation, or immediate performance, while less attention has been paid to whether gamified instructional design can support sustained learning involvement over a longer instructional period. Therefore, the present study addresses these limitations by examining gamification design elements as need-supportive instructional affordances, adopting Vallerand et al.’s tripartite classification of intrinsic motivation, and testing whether these motivational processes are associated with learning persistence in a 16-week blended digital game-based learning course.

Considering these gaps in the related literature, the present study examines gamification design elements as a higher-order latent construct rather than as separately manipulated experimental components. Specifically, this study investigates whether students’ overall perceptions of gamified instructional design—reflected in autonomy support, competence support, and relatedness support—are associated with intrinsic motivation and learning persistence. The purpose is not to determine the independent causal effect of each game element, but to clarify how an integrated gamified learning design may function as a need-supportive learning environment. By doing so, the study aims to provide a more context-sensitive explanation of how gamification can move beyond surface-level engagement and contribute to sustained motivation and persistence in blended digital game-based learning.

The theoretical contribution of this study does not lie in proposing a completely new self-determination theory pathway, but in extending and contextualizing this pathway within a blended digital game-based learning environment. While previous studies have generally confirmed that need-supportive learning environments can enhance intrinsic motivation and persistence, fewer studies have examined how gamification design elements can be interpreted as need-supportive instructional affordances that simultaneously activate autonomy support, competence support, and relatedness support. Therefore, this study advances the application of self-determination theory by translating gamification design elements into psychological need-supportive mechanisms rather than treating them merely as external rewards or technical features.

Moreover, this study further contributes to the literature by integrating Vallerand et al.’s tripartite classification of intrinsic motivation into the gamified learning context. By distinguishing intrinsic motivation to know, intrinsic motivation toward accomplishment, and intrinsic motivation to experience stimulation, the study provides a more nuanced explanation of how gamification may support different forms of autonomous learning motivation. In addition, by linking these motivational dimensions to learning persistence, this study extends prior work that mainly focuses on short-term engagement or participation. It examines whether gamified instructional design can support sustained learning involvement over a 16-week blended digital game-based course. In this sense, the study contributes to psychological theory by clarifying how need-supportive gamification may transform game-based instructional features into intrinsic motivational processes and persistent learning behavior.

## Literature review and hypothesis

Before presenting the hypotheses, it is necessary to synthesize the existing literature rather than merely list prior definitions and findings. Previous studies generally suggest that gamification can enhance motivation, engagement, and participation, but the findings are not entirely consistent. Positive effects are more likely to occur when gamification elements are meaningfully aligned with instructional goals and when they support learners’ psychological needs for autonomy, competence, and relatedness. By contrast, weak or negative effects may occur when gamification is implemented mainly as an external reward system, such as excessive reliance on points, badges, leaderboards, or rankings. In such cases, learners may focus more on reward accumulation, social comparison, or performance tracking than on the learning activity itself. Therefore, the effectiveness of gamification depends not simply on whether game elements are used, but on how they are designed, combined, and perceived within a specific learning context.

### Gamification design elements

Seaborn and Fels [51] described gamification design elements as a set of design components that can be separated from fully developed games. They stated that these elements can also be embedded into non-game activities to influence users’ experiences and behaviors. Gamification elements include points, badges, leaderboards, levels or progress indicators, Intrinsic motivation to experience stimulations, and feedback. Dichev and Dicheva [15] defined educational gamification as the integration of game design elements into educational environments. The goal is to enhance learners’ motivation and engagement. In this view, the defining feature of these elements lies not in their entertainment value, but in the use of game mechanisms to shape behavior and experience. Sailer et al. [49] stated that gamification design elements are expected to create predictable effects on specific psychological needs. Koivisto and Hamari [25] also argued that gamification elements function as motivational affordances. This is enabled by structuring feedback, goals, social interaction, and progress information. They alter users’ motivation, attitudes, and continued usage behavior. Similarly, Schobel et al. [50] characterized gamification design elements as design components that can be separated from games and implemented into learning activities. Their primary function is to reshape learners’ psychological experiences of tasks and their behavioral engagement.

This study distinguishes between concrete gamification features and learners’ perceived psychological need support. The concrete gamification features implemented in the course included points, badges, leaderboards, levels, quests and challenges, immediate feedback, progress visualization, narrative contexts, and social interaction. However, the questionnaire did not measure the separate effect of each individual feature. Instead, it measured learners’ overall perceptions of how the integrated gamified instructional design supported their basic psychological needs.

Based on self-determination theory and the work of Sailer et al. [49], perceived need-supportive gamification design was measured through three dimensions: autonomy support, competence support, and relatedness support. Autonomy support refers to the extent to which learners perceived that the gamified learning activities allowed meaningful choice, self-endorsed participation, and a sense of control. Competence support refers to the extent to which learners perceived that the gamified learning activities helped them experience task mastery, progress, feedback, and skill improvement. Relatedness support refers to the extent to which learners perceived that the gamified learning activities promoted connection, belonging, peer interaction, and collaborative participation. Therefore, the construct measured in this study was not the objective presence of gamification features, but learners’ perceived need support generated by the integrated gamified learning design.

### Intrinsic motivation

Ryan and Deci [47] argued that every individual has an inherent tendency toward active integration and growth. Contextual conditions support basic psychological needs such as autonomy, competence, and relatedness. Intrinsic motivation is more likely to be elicited and sustained in such cases. From this perspective, intrinsic motivation is not only a matter of liking an activity. However, it reflects the quality of engagement that emerges when these needs are sufficiently supported. In another study by Deci, Olafsen and Ryan [12] intrinsic motivation is defined as a specific form of autonomous motivation, in which the source of motivation can be found in the activity itself. In other words, in other words, learners engage in the activity because the process of participation itself is rewarding and motivating. Di Domenico and Ryan [14] defined intrinsic motivation as the tendency to feel Intrinsic motivation toward accomplishment, and a desire for Intrinsic motivation to experience stimulation. With this interest for Intrinsic motivation to experience stimulation individuals are willing to apply and develop their skills and knowledge. This is the case even in the absence of rewards. Fishbach and Woolley [16] emphasized that the core of intrinsic motivation lies not in a simple intrinsic–extrinsic contrast, but in the extent to which individuals psychologically integrate an activity with the goals they aim to achieve. When an activity is experienced as an end in itself, it stops functioning simply as a means to an external outcome. It becomes closely aligned with one’s personal goals, and self-concept. Similarly, Kruglanski et al. [28] pointed out that intrinsic motivation occurrs when an activity is perceived as its own worth.

Although intrinsic motivation has been discussed from different theoretical perspectives, this study adopts Ryan and Deci’s self-determination theory as the primary conceptual foundation. From this perspective, intrinsic motivation refers to learners’ autonomous willingness to engage in learning activities because the activities themselves are perceived as interesting, meaningful, challenging, and personally valuable, rather than merely as a means to obtain external rewards Ryan and Deci [47]. This definition is adopted because the present study examines how gamification design elements influence Learning Persistence through intrinsic motivation. Self-determination theory is particularly suitable for this purpose because it explains intrinsic motivation through the satisfaction of three basic psychological needs: autonomy, competence, and relatedness. These needs are closely connected to gamified instructional features such as choice, Intrinsic motivation to experience stimulation, immediate feedback, progress visualization, and social interaction. Therefore, in this study, intrinsic motivation is understood as a psychological mechanism through which gamified instructional design may strengthen learners’ awareness of learning goals, learning progress, and learning strategies.

### Learning persistence

In this study, learning persistence refers to learners’ tendency to continue participating in and completing learning activities over time, even when they encounter difficulties, distractions, reduced novelty, or motivational decline. This construct is used instead of learning awareness because the two empirical dimensions measured in this study—behavioral persistence and sustained engagement—mainly reflect learners’ continued learning behavior and ongoing involvement rather than their metacognitive awareness of learning goals, progress, or strategies. Although some prior studies have used learning awareness to describe learners’ recognition of learning goals, learning progress, learning difficulties, and learning strategies [29, 63], this study does not adopt that definition because it does not fully match the measurement structure used here. Therefore, learning persistence is a more appropriate construct label for the present study.

Learning persistence is conceptually related to engagement, but it is not identical to engagement. Engagement generally describes learners’ behavioral, emotional, and cognitive involvement in learning activities [17, 22]. Learning persistence places greater emphasis on continuity over time, especially whether learners remain involved, continue investing effort, and complete learning tasks despite obstacles. This distinction is important in blended digital game-based learning because students may show high initial participation due to the novelty of gamification, but such participation may not necessarily continue throughout a longer instructional period. Therefore, learning persistence is particularly relevant in the present 16-week course context.

This conceptualization is also consistent with research on academic persistence and online learning retention, which emphasizes continued effort, task completion, intention to remain involved, and resistance to withdrawal when learners face learning difficulties [5, 35, 36, 60, 61]. From this perspective, learning persistence is not simply a momentary state of engagement or a short-term behavioral response. Rather, it reflects learners’ ability to maintain participation, attention, effort, and task completion across the learning process.

Accordingly, this study conceptualizes learning persistence as a two-dimensional construct consisting of behavioral persistence and sustained engagement. Behavioral persistence refers to learners’ willingness and behavioral tendency to continue completing learning tasks despite difficulties or obstacles. This dimension is grounded in persistence research, which highlights perseverance, continued effort, and intention to remain in the learning process [5, 35, 36, 60, 61]. Sustained engagement refers to learners’ continued involvement, attention, and active participation throughout the learning process. This dimension is supported by student engagement literature, which views engagement as involving behavioral, emotional, and cognitive participation in learning activities [17, 22]. Together, behavioral persistence and sustained engagement reflect learners’ ability to maintain participation and involvement in learning activities over time.

### The effects of gamification design elements on intrinsic motivation

Li et al. [31], conducted a systematic review and meta-analysis of 35 intervention studies to examine the effects of gamification on students’ intrinsic motivation and basic psychological needs. They found out that gamification has a statistically significant overall effect on intrinsic motivation. Their analysis indicated that the critical factor determining this effect is whether gamification satisfies learners’ basic psychological needs. Mohanty and Christopher [41] studied the effects of three commonly used gamification elements which are points, levels, and leaderboards on intrinsic motivation and performance. These elements effectively increased behavioral engagement and task output. On the other hand, they did not any significant positive effects on intrinsic motivation nor on perceived competence. When learners interpreted these elements as tools for competition or performance tracking, motivation showed a tendency to shift toward an extrinsic orientation. This reduced interest in the task itself. Nguyen-Viet and Huong [44] investigated how gamification features influence intrinsic motivation and learning outcomes. Their findings showed that if gamification designs support immersion, give a sense of achievement, and enhance social interaction they are more likely to enhance learners’ intrinsic motivation. In another study, Xiaw and Hew [58] claimed that the effects of gamified learning elements on intrinsic motivation depend on design choices. For example, when progress systems are explicitly aligned with task-related learning goals, learners are more likely to experience Intrinsic motivation to experience stimulation and mastery. This, as a result, strengthened perceived competence. Bai et al. [3] examined the effects of gamification elements in digital courses on intrinsic motivation, Learning Persistence, and cognitive engagement. Their work focused on using points, badges, leaderboards, and Intrinsic motivation to experience stimulations. The results showed that meaningful design and immediate feedback significantly improved students’ intrinsic motivation. Moreover, learners were able to perceive progress and achievement throughout the Intrinsic motivation to experience stimulation process. When gamified designs supported autonomy and competence, learners were more likely to remain engaged out of interest and enjoy. On the other hand, designs that emphasized extrinsic rewards tended to promote only a short-term increase in their performance. Coelho et al. [10] similarly found that gamification elements, including Intrinsic motivation to experience stimulations, feedback, points, badges, and progress indicators, can enhance students’ learning motivation. More specifically, when Intrinsic motivation to experience stimulations are appropriately designed and aligned with learners’ abilities, they may stimulate learners’ interest, Intrinsic motivation toward accomplishment, and exploratory drive. Similarly, if badges and leaderboards are closely aligned with course objectives they strengthen students’ sense of achievement and willingness to participate. This, in turn, supported intrinsic motivation. However, when these elements were poorly aligned with learning content or created excessive comparison pressure, intrinsic motivation declined for some learners. Treiblmaier and Putz [53] compared gamified and non-gamified environments with respect to how intrinsic motivation influences attitudes. The results indicated that gamified environments can function as moderators. As a result, they increase the positive effects of intrinsic motivation on learning behaviors and attitudes. When gamification designs included elements that foster Intrinsic motivation toward accomplishment and Intrinsic motivation to know the predictive power of intrinsic motivation for behavioral intention was found to be stronger. These elements of Intrinsic motivation toward accomplishment and Intrinsic motivation to know include storytelling, feedback, and optional Intrinsic motivation to experience stimulations. Luarn et al. [33] also suggested that gamified reward systems (e.g., points, badges, and leaderboards) can, under certain conditions, promote intrinsic motivation. This depends on whether they support basic psychological needs. When these elements are used primarily to reinforce external outcomes, intrinsic motivation may decline. When they include choice-based badges or adjustable Intrinsic motivation to experience stimulation levels, they enhance competence and autonomy. In this case, intrinsic motivation is more likely to increase. Maryana et al. [37] compared gamified and non-gamified platforms focusing on their possible effects on student engagement, cognition, and intrinsic motivation. Their findings indicated that feedback and Intrinsic motivation to experience stimulation elements embedded in gamified designs effectively increased student engagement and motivational signals. These findings implied a facilitative effect on intrinsic motivation. On the same vein, Mekler er al. [39] studied a series of gamified courses. They reported that certain elements such as immediate feedback, well-designed Intrinsic motivation to experience stimulations, and personalized progress systems were consistently identified as the most effective. These elements led learners to perceive the learning process as worthwhile and engaging.

Taken together, these studies indicate that the relationship between gamification design elements and intrinsic motivation is conditional rather than automatic. Studies reporting positive effects usually emphasize meaningful feedback, appropriate challenge, progress visualization, autonomy-supportive choices, achievement experiences, and social interaction. These design conditions may strengthen learners’ perceived competence, autonomy, and relatedness, thereby supporting intrinsic motivation. In contrast, studies reporting limited or negative effects often involve gamification designs that rely heavily on external rewards, competition, or performance comparison. Under such conditions, learners may interpret game elements as controlling mechanisms rather than as learning support, which may weaken intrinsic motivation. Therefore, the mixed findings in previous studies can be partly explained by differences in design alignment, task meaningfulness, feedback quality, challenge level, and the balance between cooperation and competition. Based on this synthesis, the present study examines gamification design elements not as isolated rewards, but as an integrated need-supportive instructional design that may enhance intrinsic motivation.

Although prior studies have generally supported the positive relationship between gamification and intrinsic motivation, this relationship still needs to be examined in the present study for several reasons. First, previous findings suggest that the motivational effects of gamification are highly dependent on learning context, learner characteristics, and the specific combination of game elements used. Therefore, it cannot be assumed that gamification design elements will produce the same motivational effects across different instructional settings. Second, the present study does not treat gamification as a general intervention or a simple reward system. Instead, it examines a specific set of gamification design elements, including feedback, progress visualization, Intrinsic motivation to experience stimulation, achievement, and social interaction, and investigates whether these elements can support learners’ intrinsic motivation in the current digital learning context. Testing this relationship is therefore necessary as the first step in the proposed explanatory model, because intrinsic motivation is further expected to function as a psychological mechanism linking gamification design elements with Learning Persistence.

Based on the above literature, the following hypothesis is proposed:

- Hypothesis (H1): Gamification design elements have a significant positive effect on intrinsic motivation.

### The effects of intrinsic motivation on learning persistence

Zen and Xin [62] reported that students with higher levels of intrinsic motivation demonstrate significantly more sustained course participation and completion. Learning engagement partially mediated the relationship between intrinsic motivation and Learning Persistence. This indicated that intrinsic motivation first enhances learners’ interest in and focus on activities. This in turn supports persistence in learning. Similarly, Li et al. [31] emphasized that intrinsic motivation and its core psychological needs form critical psychological foundations for students’ long-term participation and sustained engagement. Sometimes, intrinsic motivation shows limited influence on short-term performance. Even when this is the case, it exerts a stable and significant effect on learners’ willingness to sustain learning activities over time. Brainbridge et al. [4] found that when students learn out of interest and a desire for self-development, they are better able to sustain learning effort for a long time. Also they are less likely to withdraw, even when tasks are challenging. Haryani et al. [19] similarly identified intrinsic motivation as a key psychological predictor of students’ continued participation in learning activities. When learners perceive the content itself as interesting and meaningful, both their persistence and effort increase significantly. Kossyva et al. [26] also noted that intrinsic motivation is the main psychological driver of sustained learning engagement. When learning activities raise interest and a sense of involvement, students are more inclined to persist. Martin and Marsh [35, 36] highlighted intrinsic motivation as an important psychological resource. It is the main factor behind learning resilience and long-term perseverance. Students with high intrinsic motivation are more likely to continue trying when they see learning difficulties. Liu and Liu [32] demonstrated that intrinsic motivation significantly enhances learners’ intentions to continue using online learning platforms. This intention widely regarded as a key antecedent of Learning Persistence. Bawa et al. [6] further argued that in online and blended learning environments, intrinsic motivation is a core factor. It supports ongoing participation and prevents drop out. When learners derive satisfaction and interest from learning activities, they are more likely to remain engaged in the long term. Youde [60, 61] found that learners’ intrinsic motivation significantly affects their willingness to continue online courses. When learning activities are considered interesting and appropriately challenging, learners sustain their engagement. Ryan and Deci [47] approached the issue from the perspective of self-determination theory. They provided a systematic explanation of how intrinsic motivation supports the maintenance of long-term behaviors. This includes sustained learning and continued effort. In turn, a solid theoretical foundation for empirical research was established.

Although the reviewed studies often use terms such as engagement, persistence, sustained participation, continued use, and course completion, these findings are directly relevant to learning persistence because they all reflect learners’ continuation of learning behavior over time. In this study, learning persistence is not defined as learners’ metacognitive awareness of learning goals, progress, or strategies. Rather, it refers to learners’ tendency to continue participating in learning activities, maintain effort, complete learning tasks, and remain involved in the course despite difficulties or reduced novelty.

From this perspective, intrinsic motivation can be expected to support learning persistence because intrinsically motivated learners are more likely to continue learning when they find the activity interesting, meaningful, challenging, or personally valuable. Such learners are also more likely to invest effort, tolerate difficulties, and remain engaged even when external rewards are limited. Therefore, prior findings on sustained engagement, continued participation, and course completion provide direct theoretical support for the relationship between intrinsic motivation and learning persistence.

Although previous studies have established that intrinsic motivation is positively related to sustained engagement, participation, and course completion, the present study extends this line of research by examining this relationship in a 16-week blended digital game-based learning context. This is important because gamified learning may generate high initial participation due to novelty or external rewards, but such participation may not necessarily continue over time. Testing H2 therefore helps clarify whether learners’ internal interest and autonomous motivation are associated with their continued effort, task completion, and sustained involvement throughout the course.

Based on the above literature, the following hypothesis is proposed:

- Hypothesis (H2) Intrinsic motivation has a significant positive effect on Learning Persistence

### The effects of gamification design elements on learning persistence

Li et al. [31] pointed out that gamification has a significant positive effect on learners’ ongoing participation and Learning Persistence. When gamification designs enhance autonomy and establish a connection, learners are more willing to remain engaged. They are also less likely to drop out. Individual achievement-related elements may have limited effects on perceived competence. Even then, gamification demonstrates stable long-term engagement. Sailer et al. [49] also provided experimental evidence. They showed that different gamification design elements influence Learning Persistence through the satisfaction of psychological needs. When elements strengthen learners’ sense of competence and relatedness, they are more likely to continue participating in tasks. Mekler et al. [39] warned that while gamification design elements can increase short-term participation, they do not necessarily promote long-term persistence. When learners interpret elements as pressure sources, engagement may decrease. Nevertheless, appropriately designed gamification can enhance Learning Persistence. Bai et al. [3] found that narrative and fantasy can significantly delay the decline in participation in online courses. Context-rich designs make students more willing to continue learning. Luarn et al. [33] demonstrated that social-oriented and immersive gamification features promote sustained engagement by enhancing intrinsic learning motivation. Treiblmaier [53] further showed that the effects of intrinsic motivation on continued use and behavioral intention are significantly amplified through gamification. This suggests that gamification strengthens the psychological processes underlying Learning Persistence. Xiao and Hew [58] reported that informational feedback increases learners’ willingness to remain engaged in online courses. Coelho et al. [10] found that gamification designs significantly increased learners’ long-term participation and course completion rates. Ma et al. [34] also showed that achievement, social interaction, and immersion significantly enhanced users’ intentions to continue using online learning platforms. This is commonly regarded as a behavioral antecedent of Learning Persistence in academic research.

Although previous studies have reported that gamification can improve sustained participation, course completion, and continued use, these findings still need further clarification in the present study because gamification does not always lead to stable persistence. Some gamified designs may increase short-term participation through novelty, rewards, or competition, but these effects may decline when learners no longer perceive the activities as meaningful or supportive. Therefore, the purpose of H3 is not simply to repeat earlier findings that gamification increases participation. Rather, this study examines whether an integrated gamified instructional design is associated with learners’ continued participation, sustained effort, task completion, and ongoing involvement throughout a 16-week blended digital game-based learning course.

From this perspective, gamification design elements may support learning persistence when they provide clear learning goals, structured challenges, immediate feedback, visible progress, and opportunities for peer interaction. These design features may help learners understand what tasks need to be completed, recognize their progress, receive timely support, and remain connected with peers during the learning process. In this way, gamification may support learning persistence not by increasing metacognitive awareness, but by creating a structured and motivating learning environment that helps learners continue participating and completing learning tasks over time.

Accordingly, testing H3 helps clarify whether perceived need-supportive gamified instructional design is positively associated with learning persistence beyond its indirect relationship through intrinsic motivation. This is important because gamification may support persistence both by strengthening learners’ internal motivation and by providing external learning structures that encourage continued engagement and task completion.

Based on the above literature, the following hypothesis is proposed:

- Hypothesis (H3): Gamification design elements have a significant positive effect on Learning Persistence.

## Research methodology

### Conceptual framework of this study

**Fig. 1:**
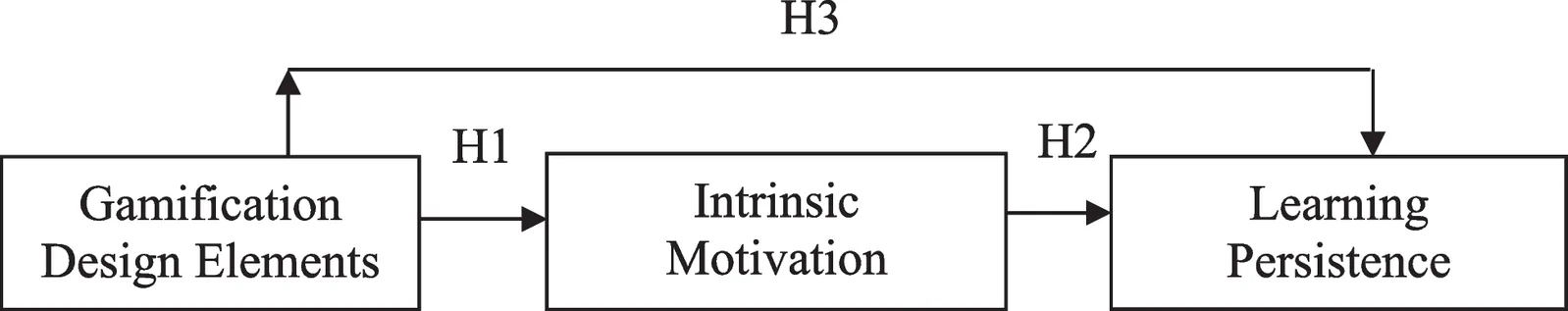
Conceptual framework of this study

The conceptual structure for this study can be seen in Fig. 1. This conceptual structure will be used to discuss the relations among Gamification Design Elements on Intrinsic Motivation and Learning Persistence

### Measurement of research variables

#### Gamification design elements

This study is based on the work of Sailer et al. [49], which is grounded in self-determination theory. According to this framework, when individuals’ basic psychological needs are satisfied in gamified learning environments, their motivation, level of engagement, and subsequent behaviors are enhanced as well. Accordingly, three key points of psychological need support were measured:

a Autonomy Support: Autonomy support refers to how much learners see their actions during system use or learning participation as self-endorsed choices.
b Competence Support: Competence support refers to learners’ perceptions of their ability to successfully complete tasks during interaction. It also includes their awareness of personal improvement and skill development.
c Relatedness Support: Relatedness support involves learners’ feelings of connection, belonging, and acceptance in relation to others while learning.

#### Intrinsic motivation

This study adopts the tripartite classification of intrinsic motivation proposed by Vallerand et al. [54], which distinguishes among intrinsic motivation to know, intrinsic motivation toward accomplishment, and intrinsic motivation to experience stimulation. This framework has been widely used in educational research to explain why learners engage in learning activities for reasons that originate from the activity itself rather than from external rewards. In the present study, this framework is applied to a blended digital game-based learning context.

First, intrinsic motivation to know refers to learners’ motivation to engage in learning because they experience pleasure and satisfaction in exploring new knowledge, understanding new ideas, and satisfying their curiosity. Second, intrinsic motivation toward accomplishment refers to learners’ motivation to participate in learning because they experience satisfaction from mastering difficult tasks, improving their abilities, and achieving learning goals. Third, intrinsic motivation to experience stimulation refers to learners’ motivation to engage in learning because they enjoy the stimulating, interesting, or emotionally satisfying experiences generated during the learning process.

Therefore, the formal analytical dimensions of intrinsic motivation in this study are intrinsic motivation to know, intrinsic motivation toward accomplishment, and intrinsic motivation to experience stimulation. Terms such as curiosity, challenge, and enjoyment are used only as explanatory descriptions to help clarify these three dimensions, not as newly proposed dimensions or independent analytical categories.

#### Learning persistence

This study conceptualizes learning persistence as learners’ tendency to continue participating in and completing learning activities in online learning environments, even when they encounter difficulties, distractions, or motivational decline. Rather than defining this construct as Learning Persistence, the revised study defines it as learning persistence because the two dimensions used in this study—behavioral persistence and sustained engagement—mainly reflect learners’ continued learning behavior and ongoing involvement in the learning process. This conceptualization is consistent with research on online learning persistence and student engagement, which emphasizes continued participation, task completion, sustained attention, and active involvement as important indicators of whether learners remain in the learning process [5, 17, 35, 36, 60, 61].

Based on this definition, learning persistence consists of two primary dimensions. First, behavioral persistence refers to learners’ willingness and behavioral tendency to continue engaging in learning activities, complete learning tasks, and remain in the course despite difficulties or obstacles. This dimension is grounded in persistence research, which highlights learners’ continued effort, perseverance, and intention to remain involved in learning even when challenges occur [5, 35, 36, 60, 61]. Second, sustained engagement refers to learners’ continued involvement, attention, and active participation throughout the learning process. This dimension is supported by student engagement literature, which views engagement as a multidimensional construct involving behavioral, emotional, and cognitive involvement in learning activities [17, 22]. Therefore, in the present study, learning persistence is measured through behavioral persistence and sustained engagement, both of which reflect learners’ ability to maintain participation and involvement in online learning activities over time.

### Research design and participants

This study adopted a one-group post-intervention survey design embedded in a 16-week blended digital game-based learning course. The study did not include a control group or a comparison between gamified and non-gamified learning conditions. Therefore, the purpose of the research design was not to establish strong causal effects, but to examine the relationships among students’ perceptions of gamification design elements, intrinsic motivation, and learning persistence after participating in the course. A total of 337 students from a university in Taichung, Taiwan participated in the present study. The course was implemented over a 16-week period, with two hours of instruction per week and 32 h in total.

The course combined face-to-face classroom instruction with digital game-based learning activities, online learning resources, platform-based tasks, and digital feedback mechanisms. Therefore, the learning environment in this study is more accurately described as a blended digital game-based learning environment rather than a fully online learning environment. After the 16-week course, data were collected through paper-based self-report questionnaires. Because all questionnaire data were collected after the intervention, the findings should be interpreted as post-intervention associations among the study variables rather than as definitive causal evidence.

The gamification design elements implemented in the course included points, badges, leaderboards, levels, quests and challenges, immediate feedback, progress visualization, narrative contexts, and social interaction. Points were used to record students’ completion of learning tasks; badges were used to recognize achievement milestones; leaderboards were used to display group or individual progress; levels were used to indicate learning stages; quests and challenges were used to structure learning tasks; immediate feedback was provided after task completion; progress visualization was used to help students observe their learning progress; narrative contexts were used to connect learning tasks with meaningful scenarios; and social interaction was encouraged through peer discussion, cooperation, and group-based activities.

In the present study, these gamification elements were implemented as an integrated instructional design package rather than as separate experimental treatments. Therefore, the questionnaire measured students’ overall perception of gamification design elements instead of estimating the independent effect of each individual element. The purpose of this study was to examine whether the overall gamified instructional design was associated with students’ intrinsic motivation and Learning Persistence. Because the study did not adopt a factorial design or compare different combinations of game elements, it cannot determine whether a specific element, such as badges, leaderboards, feedback, or challenges, independently promoted or weakened a particular psychological need. This issue is acknowledged as a limitation of the present study and may be further examined in future research through experimental designs that manipulate individual gamification elements separately.

The participants were distributed across grade levels as follows: 122 first-year students, 113 s-year students, and 102 third-year students. A total of 144 male students and 193 female students were included in the study.

### Course context and instructional consistency

To provide clearer contextual information about the learning environment, this study further describes the course context, instructional method, and instructor arrangement. The 16-week intervention was implemented in an Educational Technology course offered to undergraduate students at a university in Taichung, Taiwan. The course aimed to help students understand key concepts, apply course knowledge to learning tasks, and complete individual and collaborative activities through a blended digital game-based learning format. The learning content was organized into weekly instructional units, and each unit included teacher explanation, digital learning tasks, platform-based activities, feedback, and gamified participation mechanisms.

The instructional method combined face-to-face teaching, task-based learning, digital game-based activities, peer discussion, group cooperation, and formative feedback. At the beginning of each weekly session, the instructor introduced the learning objectives, key concepts, and task requirements. Students then completed individual or group-based quests, challenges, or platform-supported learning activities related to the course content. After completing the tasks, students received feedback from the instructor, peers, or the digital platform. Gamified mechanisms such as points, badges, levels, leaderboards, progress visualization, and challenge tasks were used to make learning goals, progress, and achievement more visible.

Instructional consistency was maintained throughout the 16-week course. The same instructor was responsible for course design, teaching delivery, task explanation, feedback, and implementation of the gamification rules. The instructor followed a fixed weekly teaching schedule, common task structure, consistent participation rules, and the same assessment criteria during the intervention. This arrangement helped reduce possible differences caused by instructor variation, teaching style, task explanation, or feedback standards. Therefore, the findings of this study should be interpreted within this specific blended digital game-based learning environment, in which the instructional content, gamified task structure, and instructor implementation were kept consistent.

### Gamified course design and implementation

To improve the replicability of the intervention, this study provides a detailed description of how the gamified course was structured and implemented. The 16-week course was designed as a blended digital game-based learning course. Each week included face-to-face instruction, digital learning tasks, platform-based activities, and feedback mechanisms. The gamified system was not implemented as a separate game unrelated to the course. Rather, game elements were embedded into regular learning tasks to support goal orientation, feedback, progress monitoring, achievement recognition, and peer interaction.

The gamified course included nine main design elements: points, badges, leaderboards, levels, quests and challenges, immediate feedback, progress visualization, narrative contexts, and social interaction. Points were awarded when students completed weekly learning tasks, participated in classroom or platform-based activities, submitted assignments, or completed challenge tasks. Badges were used to recognize achievement milestones, such as completing a set of tasks, maintaining continuous participation, or demonstrating improvement. Leaderboards displayed individual or group progress at selected points during the course, but they were used mainly to provide progress information rather than to create excessive competition. Levels represented the staged structure of the course and indicated students’ progression from basic tasks to more complex learning challenges. Quests and challenges were used to organize learning activities into goal-based tasks that required students to apply course content, solve problems, or complete collaborative activities.

Immediate feedback was provided after task completion through teacher comments, platform responses, peer discussion, or visible task results. Progress visualization was used to help students monitor their learning status, accumulated points, completed tasks, badges earned, and current level. Narrative contexts were used to connect weekly learning tasks with meaningful learning scenarios, so that students could understand the purpose and relevance of each task. Social interaction was encouraged through peer discussion, group cooperation, shared tasks, and collaborative problem-solving activities.

The participation rules were explained to students at the beginning of the course. Students were informed about how points could be earned, how badges could be obtained, how levels were structured, and how challenge tasks should be completed. Participation in gamified activities was connected to regular course learning tasks. However, the gamified rewards themselves were not directly converted into formal course grades. Points, badges, and leaderboards were used as formative feedback and motivational indicators rather than as summative assessment scores. Formal course evaluation remained based on regular course requirements, such as attendance, assignments, learning tasks, and final performance. This design was adopted to reduce the risk that students would participate only for external rewards and to encourage them to interpret gamification as learning support rather than as grade competition.

For replicability, the overall structure of the gamified course can be summarized as follows. In the early weeks, students became familiar with the learning platform, task rules, point system, and basic challenges. In the middle weeks, students participated in more complex quests, group-based tasks, feedback activities, and progress-monitoring activities. In the later weeks, students completed integrative challenges that required them to apply what they had learned, reflect on their progress, and maintain participation until the end of the course. Screenshots of the learning platform, examples of badges, sample challenge tasks, and a sample progress dashboard can be provided in an appendix to illustrate how the gamified system was implemented.

### Research methods

#### Literature analysis

A literature analysis was conducted to collect and examine relevant sources, such as academic journals and scholarly articles. Through review and analysis of existing studies, the characteristics of the research topic were explored, key viewpoints were synthesized, and the research problem was defined. Through this process a comprehensive understanding of the research theme was obtained.

#### Questionnaire development and survey procedure

A questionnaire survey method was used in this study to collect standardized responses from the participants. The questionnaire was developed based on the research framework and relevant validated scales from previous studies. The questionnaire consisted of four sections: demographic information, gamification design elements, intrinsic motivation, and Learning Persistence. The items for gamification design elements were developed with reference to prior gamification research, including points, badges, leaderboards, levels, quests and challenges, immediate feedback, progress visualization, narrative contexts, and social interaction. The items for intrinsic motivation were adapted from the tripartite classification proposed by Vallerand et al. [54], including intrinsic motivation to know, intrinsic motivation toward accomplishment, and intrinsic motivation to experience stimulation. The items for Learning Persistence were developed based on metacognitive awareness and self-regulated learning literature, focusing on learners’ recognition of learning goals, learning progress, learning difficulties, and learning strategies.

To ensure content validity, the initial version of the questionnaire was reviewed by experts in educational technology, instructional design, and educational psychology. The experts examined whether the items were consistent with the theoretical definitions of each construct and whether the wording was clear and appropriate for university students. Based on their suggestions, ambiguous or repetitive items were revised. A pilot test was then conducted before the formal survey to examine the clarity of the questionnaire items and the appropriateness of the response format. The questionnaire was designed using a five-point Likert scale, ranging from 1= strongly disagree to 5 = strongly agree.

After the 16-week blended digital game-based learning course, the formal questionnaire was administered face to face. This approach helped ensure that participants clearly understood the questionnaire instructions and completed the survey independently. After data collection, statistical analyses were conducted to examine the reliability and validity of the scales. Exploratory factor analysis was used to examine the construct validity of the questionnaire, including the factor structure of gamification design elements, intrinsic motivation, and Learning Persistence. The Kaiser–Meyer–Olkin value and Bartlett’s test of sphericity were used to assess whether the data were suitable for factor analysis. Internal consistency reliability was evaluated using Cronbach’s alpha. These procedures were used to ensure that the measurement scales had acceptable validity and reliability before hypothesis testing.

## Analysis and discussion

### Exploratory factor analysis and reliability analysis

To examine the empirical dimensionality and internal consistency of the measurement scales, this study conducted exploratory factor analysis (EFA) and reliability analysis. EFA was used because the questionnaire items were adapted from previous theoretical frameworks and revised to fit the blended digital game-based learning context of the present study. Therefore, it was necessary to examine whether the adapted items formed the expected factor structure in the current sample. Principal axis factoring was used as the extraction method, and varimax rotation was applied to obtain a clearer factor structure. Factors were retained based on eigenvalues greater than 1.00, theoretical interpretability, and item loadings on the intended factor. Cronbach’s alpha was used to assess the internal consistency reliability of each factor.

The results of the EFA are presented in Table 1. For the gamification design elements scale, three factors were extracted: Autonomy Support, Competence Support, and Relatedness Support. These dimensions were theoretically grounded in self-determination theory, which emphasizes autonomy, competence, and relatedness as three basic psychological needs. The first factor was Autonomy Support (eigenvalue = 2.528, α = 0.86), the second factor was Competence Support (eigenvalue = 2.231, α = 0.88), and the third factor was Relatedness Support (eigenvalue = 1.863, α = 0.87). These three factors accounted for a cumulative explained variance of 75.864%.

**Table 1:** Factor analysis results

Variable	Factor Dimension	Eigenvalue	Α	Cumulative Explained Variance (%)
Gamification Design Elements	Autonomy Support	2.528	0.86	75.864
	Competence Support	2.231	0.88	
	Relatedness Support	1.863	0.87	
Intrinsic Motivation	Intrinsic motivation to know	3.152	0.89	79.762
	Intrinsic motivation toward accomplishment	2.745	0.91	
	Intrinsic motivation to experience stimulation	1.964	0.92	
Learning Persistence	Behavioral Persistence	4.055	0.90	80.583
	Sustained Engagement	3.317	0.94	

For the intrinsic motivation scale, three factors were extracted: intrinsic motivation to know, intrinsic motivation toward accomplishment, and intrinsic motivation to experience stimulation. These three dimensions followed Vallerand et al.’s [54] tripartite classification of intrinsic motivation. The first factor was intrinsic motivation to know (eigenvalue = 3.152, α = 0.89), the second factor was intrinsic motivation toward accomplishment (eigenvalue = 2.745, α = 0.91), and the third factor was intrinsic motivation to experience stimulation (eigenvalue = 1.964, α = 0.92). The cumulative explained variance of these three factors reached 79.762%.

For the Learning Persistence scale, two factors were extracted: Behavioral Persistence and Sustained Engagement. The first factor was Behavioral Persistence (eigenvalue = 4.055, α = 0.90), and the second factor was Sustained Engagement (eigenvalue = 3.317, α = 0.94). The two factors jointly explained 80.583% of the total variance.

Overall, the extracted factors were consistent with the theoretical structure adopted in this study, and all Cronbach’s alpha values exceeded the commonly accepted threshold of 0.70. These results indicate that the measurement scales had acceptable construct validity and internal consistency reliability for subsequent hypothesis testing.

### Correlation analysis

As shown in Table 2, gamification design elements, intrinsic motivation, and Learning Persistence were all significantly correlated. These results provide preliminary support for Hypotheses H1, H2, and H3.

**Table 2:** Correlation analysis

Research Construct	α	Gamification Design Elements	Intrinsic Motivation	Learning Persistence
Gamification Design Elements	0.88			
Intrinsic Motivation	0.91	0.24**		
Learning Persistence	0.92	0.29**	0.35**	

** *p* < 0.01

### Structural equation model fit indices

Structural equation modeling (SEM) uses factor analysis and path analysis which are considered as traditional statistical methods. SEM also incorporates the concept of simultaneous equations from econometrics. SEM allows the simultaneous estimation of multiple factors and multiple causal pathways. Model fit evaluation is commonly conducted from three perspectives: preliminary fit criteria, overall model fit, and the fit of the model’s internal structure.

The results of the data analysis are summarized as follows. The model is evaluated in terms of its preliminary fit, internal structural fit, and overall fit.

As shown in the full model analysis results in Table 3, the preliminary fit criteria indicate that all three factor dimensions of gamification design elements (autonomy support, competence support, and relatedness support) significantly explained the higher-order construct of gamification design elements (t > 1.96, *p* < 0.05). Similarly, all three factor dimensions of intrinsic motivation (Intrinsic motivation to know, Intrinsic motivation toward accomplishment, and Intrinsic motivation to experience stimulation) significantly explained intrinsic motivation (t > 1.96, *p* < 0.05). In addition, both factor dimensions of Learning Persistence (behavioral persistence and sustained engagement) significantly explained the construct of Learning Persistence (t > 1.96, *p* < 0.05). These results indicate that the overall model demonstrates satisfactory preliminary fit.

**Table 3:** Results of the overall structural equation model analysis

Evaluation Category	Parameter/Evaluation Standard		Result
Preliminary Fit	Gamification Design Elements	Autonomy Support	0.702**
		Competence Support	0.688**
		Relatedness Support	0.691**
	Intrinsic Motivation	Intrinsic motivation to know	0.713**
		Intrinsic motivation toward accomplishment	0.737**
		Intrinsic motivation to experience stimulation	0.725**
	Learning Persistence	Behavioral Persistence	0.768**
		Sustained Engagement	0.755**

** *p* < 0.01

As shown in Table 4, with respect to the internal structural fit, gamification design elements exhibited a significant positive relationship with intrinsic motivation (0.321, *p* < 0.01). Intrinsic motivation also showed a significant positive relationship with Learning Persistence (0.383, *p* < 0.01), and gamification design elements were significantly and positively related to Learning Persistence as well (0.416, *p* < 0.01). These findings indicate that Hypotheses H1, H2, and H3 are all supported.

**Table 4:** Results of the overall structural equation model analysis

Evaluation Category	Parameter/Evaluation Standard	Result
Internal Structural Fit	Gamification Design Elements → Intrinsic Motivation	0.321**
	Intrinsic Motivation → Learning Persistence	0.383**
	Gamification Design Elements → Learning Persistence	0.416**

** *p* <.01

### Mediation analysis

To further examine whether intrinsic motivation mediated the relationship between perceived need-supportive gamification design and learning persistence, a mediation analysis was conducted based on the structural equation model. The indirect effect was calculated by multiplying the path coefficient from perceived need-supportive gamification design to intrinsic motivation by the path coefficient from intrinsic motivation to learning persistence. The result showed that the indirect effect was 0.123, calculated as 0.321 × 0.383. The direct effect of perceived need-supportive gamification design on learning persistence remained significant at 0.416. Therefore, the total effect was 0.539.

These results indicate that intrinsic motivation partially mediated the relationship between perceived need-supportive gamification design and learning persistence. In other words, perceived need-supportive gamification design was associated with learning persistence both directly and indirectly through intrinsic motivation. The direct pathway suggests that gamified instructional design may support students’ persistence through structured tasks, feedback, progress visualization, and social interaction. The indirect pathway suggests that gamified instructional design may also support persistence by strengthening students’ intrinsic motivation. Because both the direct effect and the indirect effect were present, the mediation pattern should be interpreted as partial mediation rather than full mediation.

As shown in Table 5, the overall model fit was evaluated using multiple fit indices. The χ^2^/df value was 1.683, which was below the commonly recommended threshold of 3.00. The GFI and AGFI values were 0.974 and 0.941, respectively, both exceeding the recommended criterion of 0.90. The RMR value was 0.004, indicating a low residual value. In addition, the CFI value was 0.963 and the TLI value was 0.948, suggesting acceptable incremental model fit. The RMSEA value was 0.045 and the SRMR value was 0.032, both below the recommended threshold of 0.08. Taken together, these indices suggest that the proposed model achieved acceptable model fit. However, the model fit results should be interpreted as supporting the plausibility of the proposed model rather than as evidence of strong explanatory power.

**Table 5:** Results of the overall structural equation model analysis

Overall Model Fit	X^2^/Df	1.683
	GFI	0.974
	AGFI	0.941
	RMR	0.004
	CFI	0.963
	TLI	0.948
	RMSEA	0.045
	SRMR	0.032

In addition to model fit indices, the standardized path coefficients and mediation estimates were used to evaluate the magnitude of the relationships in the proposed model. The standardized coefficient from gamification design elements to intrinsic motivation was 0.321, the coefficient from intrinsic motivation to learning persistence was 0.383, and the coefficient from gamification design elements to learning persistence was 0.416. These values indicate statistically significant and meaningful positive associations, but they should be interpreted as moderate rather than strong effects. The mediation analysis further showed an indirect effect of 0.123, a direct effect of 0.416, and a total effect of 0.539. Therefore, the findings provide preliminary explanatory support for the proposed model, but they should not be interpreted as evidence of strong explanatory power or definitive causal influence.

## Discussion

This study examined the relationships among perceived need-supportive gamification design, intrinsic motivation, and learning persistence in a 16-week blended digital game-based learning course. The findings provide preliminary empirical support for the proposed model. Students who perceived stronger need support from the gamified instructional design tended to report higher levels of intrinsic motivation and stronger learning persistence. Intrinsic motivation was also positively associated with learning persistence and partially mediated the relationship between gamified design and learning persistence. These findings suggest that gamification should not be understood merely as the addition of visible game elements, such as points, badges, leaderboards, or challenges. Rather, its educational value depends on whether these elements are perceived by learners as supporting autonomy, competence, and relatedness.

The first important finding is that perceived need-supportive gamification design was positively associated with intrinsic motivation. This result can be interpreted through self-determination theory. From this perspective, learning environments are more likely to foster intrinsic motivation when they support learners’ needs for autonomy, competence, and relatedness. In the present study, gamified instructional design may have supported autonomy by giving learners clearer choices, task routes, and opportunities for self-directed participation. It may have supported competence by providing feedback, visible progress, achievable challenges, and recognition of task completion. It may also have supported relatedness by encouraging peer interaction, group participation, and collaborative problem-solving. Therefore, the motivational value of gamification appears to come not only from making learning more enjoyable, but from reshaping learners’ psychological experience of the learning environment.

This interpretation helps explain why prior studies have reported inconsistent findings regarding gamification. Some studies have found that gamification increases motivation, engagement, and participation, whereas others have reported weak, unstable, or even negative effects. The present findings suggest that these inconsistencies may be related to differences in design quality and psychological meaning. When gamification is meaningfully connected to instructional goals and supports learners’ sense of choice, mastery, feedback, and social connection, it is more likely to strengthen intrinsic motivation. In contrast, when gamification relies mainly on external rewards, rankings, or competition, learners may focus on reward accumulation or social comparison rather than on learning itself. Under such conditions, game elements may be experienced as controlling or evaluative rather than autonomy-supportive. This may explain why points, badges, and leaderboards sometimes increase short-term participation but fail to produce stable intrinsic motivation.

The second important finding is that intrinsic motivation was positively associated with learning persistence. This finding suggests that sustained learning is not only a matter of behavioral participation, but also a psychological process. Learners who participate because they find the activity interesting, meaningful, challenging, or personally valuable are more likely to continue investing effort over time. In a blended digital game-based course, students may initially participate because the activities are novel or because game elements provide external stimulation. However, novelty effects may decline as the course continues. Under such conditions, intrinsic motivation becomes especially important because it helps learners continue participating even when external stimulation decreases. Thus, intrinsic motivation may function as an internal psychological resource that supports continued effort, task completion, and sustained involvement throughout the learning process.

This finding also clarifies the meaning of learning persistence in the present study. Learning persistence differs from momentary engagement. Engagement describes learners’ involvement in learning activities, whereas learning persistence emphasizes whether learners continue participating, maintain effort, and complete tasks over time despite difficulties or reduced novelty. The positive relationship between intrinsic motivation and learning persistence suggests that learners are more likely to remain involved when they experience learning as internally meaningful rather than merely externally required. This supports the argument that digital and gamified learning should not only aim to increase initial engagement, but should also cultivate internal motivation that can sustain learning across a longer instructional period.

The third important finding is that perceived need-supportive gamification design was directly associated with learning persistence. This result suggests that gamification may support persistence not only through intrinsic motivation, but also through the structure it provides to the learning environment. For example, clear quests and challenges may help students understand what tasks need to be completed. Immediate feedback may reduce uncertainty and help learners correct mistakes in a timely manner. Progress visualization may help students recognize their current learning status and remaining goals. Badges and levels may provide achievement signals that help students perceive progress. Social interaction may create peer support and a sense of shared participation. These design features may make continued participation easier and more structured, even when learners’ internal motivation fluctuates.

The partial mediation result further deepens this interpretation. The indirect effect of perceived need-supportive gamification design on learning persistence through intrinsic motivation indicates that gamified instructional design may support persistence by strengthening learners’ internal motivation. At the same time, the direct effect remained significant, suggesting that intrinsic motivation did not fully explain the relationship between gamification and learning persistence. This means that gamification may operate through both motivational and structural pathways. The motivational pathway works through intrinsic motivation, while the structural pathway works through task organization, feedback, progress monitoring, and peer interaction. Therefore, effective gamification should be understood as a psychologically supportive instructional structure rather than a simple motivational decoration.

These findings provide stronger psychological insight into the role of gamification in learning. The same game element may produce different outcomes depending on how learners interpret it. A leaderboard, for example, may support competence if it helps learners understand their progress, but it may undermine motivation if it creates excessive comparison pressure. A badge may support persistence if it recognizes meaningful achievement, but it may become a superficial reward if it is disconnected from learning goals. Similarly, challenges may stimulate interest when they are appropriately difficult, but may discourage learners if they are too difficult or poorly supported. Therefore, the psychological function of game elements is more important than their surface form. The key question is not whether gamification elements are present, but whether they are experienced as supportive, meaningful, and connected to learning.

The findings also extend the application of self-determination theory in gamified learning contexts. Rather than treating gamification as a general intervention, this study conceptualizes gamified design as perceived need support. This provides a more nuanced explanation of how gamification may influence learning outcomes. Game elements are not assumed to be motivating by themselves. Instead, they are understood as instructional affordances that may support or fail to support psychological needs depending on how they are designed and implemented. This perspective helps connect gamification research with motivational psychology and explains why gamification effects are conditional rather than automatic.

In addition, the use of Vallerand et al.’s three-dimensional view of intrinsic motivation provides a more detailed understanding of learners’ motivational experience. In a gamified learning environment, students may be motivated by the pleasure of knowing new content, the satisfaction of mastering difficult tasks, or the stimulating experience of participating in game-like learning activities. These different forms of intrinsic motivation help explain why gamification may influence learners in more than one way. A well-designed gamified course may stimulate curiosity, support accomplishment, and create enjoyable learning experiences at the same time. This multidimensional interpretation prevents intrinsic motivation from being treated as a single general feeling and provides a richer psychological explanation of learners’ sustained participation.

The findings also have practical implications for blended digital game-based learning. Educators should avoid using gamification only as a reward system. Points, badges, and leaderboards may attract attention, but they should be connected to meaningful learning goals, clear feedback, and opportunities for improvement. Challenges should be appropriately difficult and should allow students to experience progress and mastery. Feedback should be timely and informative rather than merely evaluative. Progress visualization should help learners monitor their learning status and recognize their improvement. Social interaction should be designed to support collaboration and belonging rather than excessive competition. In this way, gamification can function as a need-supportive learning environment that encourages both intrinsic motivation and learning persistence.

The findings should nevertheless be interpreted with caution. This study adopted a one-group post-intervention survey design, and all data were collected through self-report questionnaires after the 16-week course. Therefore, the results should be understood as evidence of associations among perceived need-supportive gamification design, intrinsic motivation, and learning persistence rather than as definitive causal evidence. In addition, the gamification elements were implemented as an integrated instructional package. The study therefore cannot determine the independent effect of each individual element, such as points, badges, leaderboards, feedback, or challenges. Future research should use longitudinal, quasi-experimental, or factorial experimental designs to examine how specific gamification elements influence autonomy, competence, relatedness, intrinsic motivation, and learning persistence over time.

Overall, this study suggests that gamification is most educationally valuable when it is designed as a psychologically supportive instructional structure. Gamification may fail when it relies mainly on external rewards or competition, but it may be more effective when it supports learners’ autonomy, competence, and relatedness. The present findings indicate that perceived need-supportive gamification design is positively associated with intrinsic motivation and learning persistence, and that intrinsic motivation partially explains this relationship. Therefore, gamification should not be viewed simply as a way to make learning fun. Rather, it should be understood as a design approach that can transform learning tasks into more meaningful, structured, and psychologically supportive experiences.

This study examined the relationships among gamification design elements, intrinsic motivation, and learning persistence in a blended digital game-based learning course. The findings provide preliminary empirical support for the proposed model, suggesting that students’ perceptions of gamified instructional design were positively associated with intrinsic motivation and learning persistence. However, because the measurement scales were adapted to the present instructional context and examined primarily through exploratory factor analysis, the results should be interpreted cautiously. The purpose of this discussion is therefore not to claim that the measurement model has been fully established, but to explain how the current findings provide initial evidence for the theoretical relationships proposed in this study.

Before interpreting the structural relationships, it is necessary to clarify the measurement foundation of the study. Exploratory factor analysis and reliability analysis were conducted to examine the dimensionality and internal consistency of the measurement scales. The gamification design elements scale was differentiated into three factors: autonomy support, competence support, and relatedness support. This structure is consistent with self-determination theory, which emphasizes autonomy, competence, and relatedness as basic psychological needs. In the present study, this finding suggests that students did not perceive gamification merely as a collection of external rewards or technical features. Rather, they appeared to perceive gamified learning design in terms of the psychological support it provided during learning. This result supports the view that gamification may be more meaningful when it is designed to support learners’ choices, perceived mastery, and social connection.

The intrinsic motivation scale also showed a three-factor structure: intrinsic motivation to know, intrinsic motivation toward accomplishment, and intrinsic motivation to experience stimulation. These dimensions follow Vallerand et al.’s tripartite classification of intrinsic motivation. The results suggest that intrinsic motivation in the present blended digital game-based learning context can be understood as a multidimensional construct. Students may be intrinsically motivated because they enjoy understanding new knowledge, because they feel satisfaction from completing challenging tasks, or because they experience stimulation and interest during the learning process. This finding is important because digital game-based learning does not motivate students only by making learning “fun.” Instead, motivation may also arise from exploration, accomplishment, challenge, and meaningful learning experiences. Therefore, the findings provide initial support for using Vallerand et al.’s framework to examine intrinsic motivation in a blended digital game-based learning context.

The construct originally labeled as Learning Persistence was reconsidered in this study. Because the factor analysis identified two dimensions—behavioral persistence and sustained engagement—the construct is more accurately described as learning persistence. Behavioral persistence refers to students’ tendency to continue completing learning tasks despite difficulties, while sustained engagement refers to their continued involvement, attention, and participation during the learning process. These two dimensions mainly reflect learners’ ongoing participation and continued effort rather than their metacognitive awareness of learning goals, progress, or strategies. Therefore, renaming the construct as learning persistence helps align the conceptual definition with the empirical measurement structure. This revision also avoids conceptual overlap between Learning Persistence, engagement, and course completion.

The correlation results showed significant positive relationships among gamification design elements, intrinsic motivation, and learning persistence. Specifically, gamification design elements were positively correlated with intrinsic motivation and learning persistence, while intrinsic motivation was also positively correlated with learning persistence. These results provide preliminary evidence that students who perceived stronger gamified design support also reported higher levels of intrinsic motivation and stronger learning persistence. In addition, the correlation between intrinsic motivation and learning persistence was stronger than the correlation between gamification design elements and learning persistence. This pattern suggests that students’ internal motivation may be an important psychological mechanism in sustaining participation in a blended digital game-based learning environment.

The structural equation modeling results further supported the proposed relationships. Gamification design elements had a significant positive effect on intrinsic motivation, indicating that when students perceived the gamified course as supporting autonomy, competence, and relatedness, they were more likely to develop intrinsic motivation. This finding is consistent with self-determination theory, which argues that learning environments are more likely to foster intrinsic motivation when they support learners’ psychological needs. In the present study, gamification design elements such as feedback, progress visualization, challenges, achievement indicators, and social interaction may have helped students experience greater control, competence, and connection during learning. Therefore, the positive relationship between gamification design elements and intrinsic motivation suggests that gamification can function as a motivational support mechanism when it is meaningfully integrated into instructional design.

Intrinsic motivation also had a significant positive effect on learning persistence. This result indicates that students who engaged in learning because of interest, accomplishment, or stimulating learning experiences were more likely to maintain participation and continue investing effort in the course. This finding supports the theoretical argument that intrinsic motivation contributes not only to immediate learning interest but also to sustained learning behavior. In a blended digital game-based course, students may encounter cognitive demands, task difficulties, or reduced novelty over time. Under such conditions, intrinsic motivation may help students continue participating because the learning activity itself remains meaningful, interesting, or personally satisfying. Therefore, intrinsic motivation can be understood as an important psychological resource that supports persistence beyond short-term participation.

Gamification design elements also had a direct positive effect on learning persistence. This result suggests that gamified instructional design may support students’ continued participation not only indirectly through intrinsic motivation, but also directly through course structure, task organization, feedback, and social interaction. For example, progress visualization may help students recognize how far they have advanced, immediate feedback may reduce uncertainty during task completion, and quests or challenges may provide clear learning goals. Social interaction may also encourage students to remain involved through peer support and collaborative participation. These elements may create a learning structure that helps students continue engaging with the course even when motivation fluctuates. However, this finding should be interpreted as the effect of the integrated gamified instructional design rather than the independent effect of any single gamification element.

The overall model fit indices indicated that the proposed structural model had an acceptable fit to the data. The χ^2^/df, RMR, GFI, and AGFI values all met commonly accepted standards, suggesting that the hypothesized relationships were reasonably consistent with the observed data. Nevertheless, these results should be interpreted as preliminary support rather than conclusive validation. Although exploratory factor analysis and reliability analysis provided initial evidence for the factor structure and internal consistency of the scales, future research should further examine the measurement model through confirmatory factor analysis, convergent validity, discriminant validity, and cross-sample validation. Such additional analyses would strengthen the psychometric foundation of the constructs and provide stronger evidence for the stability of the proposed model.

Taken together, the findings suggest that gamified learning environments may be most effective when they support learners’ psychological needs, foster intrinsic motivation, and provide structures that encourage continued participation. The results indicate that gamification should not be understood simply as the addition of points, badges, or leaderboards. Instead, effective gamified instructional design should be connected to autonomy support, competence support, and relatedness support. When students perceive gamification as meaningful support for learning rather than as external control or competition, they may become more intrinsically motivated and more willing to persist in learning activities.

This study contributes to the literature in several ways. First, it applies self-determination theory to examine how gamification design elements are related to intrinsic motivation in a blended digital game-based learning context. Second, it uses Vallerand et al.’s three-dimensional classification to examine intrinsic motivation more specifically, rather than treating it as a single general construct. Third, by revising the outcome construct as learning persistence, the study provides a more conceptually consistent explanation of how behavioral persistence and sustained engagement can be understood in relation to gamification and intrinsic motivation. These contributions are especially relevant for higher education contexts in which digital and game-based learning tools are increasingly used to maintain students’ participation over an extended instructional period.

Despite these contributions, several limitations should be acknowledged. First, the measurement scales were adapted to the present instructional context, and the current evidence was mainly based on exploratory factor analysis and Cronbach’s alpha. Future studies should conduct confirmatory factor analysis and examine composite reliability, average variance extracted, and discriminant validity to further validate the measurement model. Second, the gamification design elements were implemented as an integrated instructional package. Therefore, this study cannot determine the separate effects of individual elements such as badges, leaderboards, feedback, challenges, or social interaction. Future research may use factorial experimental designs or component-based intervention designs to examine whether specific gamification elements independently support or weaken autonomy, competence, and relatedness. Third, because the study was conducted in one university context, the generalizability of the findings may be limited. Future studies should examine whether the proposed model is stable across different disciplines, learner populations, course formats, and cultural contexts.

In summary, this study provides preliminary evidence that gamification design elements are positively associated with intrinsic motivation and learning persistence in a blended digital game-based learning course. The findings suggest that the motivational value of gamification depends not merely on the presence of game elements, but on whether these elements support learners’ psychological needs and encourage sustained involvement in learning. By adopting a more cautious interpretation of the results and clarifying the measurement structure of the constructs, this study offers a foundation for future research on how gamified instructional design can support students’ motivation and persistence in digital learning environments.

## Conclusion

This study used a game-based course and an experimental research design with structural equation modeling to examine the relationships among gamification design elements, intrinsic motivation, and Learning Persistence. The theoretical model demonstrates strong fit and explanatory power. Moreover, all measurement dimensions exhibited stable and satisfactory reliability and validity, indicating that the study is methodologically rigorous and theoretically sound.

Gamification design confirms it goes beyond surface mechanisms like points, badges, or leaderboards by addressing learners’ basic psychological needs to shape learning experiences. When designs offer meaningful choices, clear and informative feedback, and appropriate opportunities for social interaction, they support engagement. With regard to intrinsic motivation, the results suggest that intrinsic motivation to know, intrinsic motivation toward accomplishment, and intrinsic motivation to experience stimulation can be used to understand students’ motivational experiences in a blended digital game-based learning context. Accordingly, the concept of motivation is multifaceted: it includes Intrinsic motivation to know, interest in new knowledge and a sense of accomplishment as a result of being presented challenging tasks. Courses that focus solely on entertainment value may struggle to sustain motivation over time. Well-designed Intrinsic motivation to experience stimulations are essential for ongoing exploration and learning.

This study shows that Learning Persistence includes outcome-oriented and process-oriented dimensions. Behavioral persistence means completing tasks despite difficulties. Sustained engagement means maintaining participation and effort throughout the entire learning process. This distinction offers a nuanced viewand prevents oversimplification by using single indicators such as course completion rates or short-term performance outcomes.

The results reveal that gamification design boosts intrinsic motivation, which enhances larning awareness. It also has a direct effect on Learning Persistence. These findings indicate that gamification influences persistence via two pathways. First, it indirectly promotes sustained engagement in learning over time. Second, through well-structured learning environments and effective feedback, it reduces the likelihood of dropout. Taken together, the overall findings suggest that in merely adding technologies is not enough to ensure long-term learner engagement. Stable intrinsic motivation and a supportive learning environment are key to Learning Persistence. Gamification based on self-determination theory that supports autonomy, competence, and relatedness, short-term learning interest into long-term Learning Persistence.

This study shows that the integration of gamification into learning is a strategic approach that influences learning processes through psychological mechanisms. When instructional design integrates technological applications, psychological support, and well-structured learning processes, it fosters engagement and growth over time. These conclusions address key Intrinsic motivation to experience stimulations in higher education’s digital transformation and offer practical directions for future research and practice in gamified instruction.

## Recommendations

The findings of this study indicate that gamification can enhance students’ Learning Persistence both indirectly—by strengthening intrinsic motivation—and directly. Based on these results, the following practical recommendations are proposed.

### Reframing gamified course design around psychological need support

Gamification design should go beyond surface-level mechanisms like points, badges, or leaderboards. It should support learners’ psychological needs. For autonomy support, course designers can provide multiple task pathways and meaningful choices. This allows students to select learning approaches that align with their interests, abilities, or learning styles. This not only helps reduce learning pressure but also enhances learners’ sense of control over the learning process. As a result it fosters intrinsic motivation. As for competence support, particular attention should be given to the design of timely and specific feedback. Making learners’ progress visible can strengthen their sense of competence and prevent disengagement caused by a lack of perceived progress. Incorporating cooperative and social gamification features can enhance relatedness support by helping learners feel supported by peers and connected through shared goals during the learning process.

### Positioning the development of intrinsic motivation as the core of long-term learning strategies

In practice, intrinsic motivation should be seen as a key focus of long-term learning strategies, rather than just a byproduct of short-term performance. With respect to Intrinsic motivation to know, instructors can make learning activities more appealing through techniques like contextualization, storytelling, or role-playing, enabling students to feel positive emotions while learning. Regarding Intrinsic motivation toward accomplishment, course content can incorporate problem-based or inquiry-oriented approaches, intentionally withholding complete answers to promote active student exploration. When it comes to Intrinsic motivation to experience stimulation, educators should not equate Intrinsic motivation to experience stimulation with excessive difficulty or workload. Instead, the aim is to offer the right level of Intrinsic motivation to experience stimulation by adapting task difficulty in real time, keeping learners engaged in a zone where effort is required but success is still achievable—thus preventing frustration from overly difficult tasks and boredom from tasks that are too easy.

#### Using learning persistence as a key indicator of course quality

Educational institutions should incorporate Learning Persistence into course evaluations and teaching effectiveness metrics. Using platform data and survey instruments, institutions can simultaneously capture students’ participation frequency, intentions to continue learning, and actual completion behaviors throughout the course. These indicators can serve as evidence for course improvement. These metrics help instructors identify early signs of potential disengagement and offer timely learning support.

#### Enhancing teachers’ pedagogical literacy in gamification design

Teachers’ pedagogical skills and design abilities play a critical role in effective gamification. Educational institutions should provide systematic training and support to help instructors understand ethical considerations and learn how to meaningfully align gamification with course objectives. Training programs may focus on using gamification as a learning support tool, designing gamified activities that foster intrinsic motivation, and evaluating Learning Persistence. Strengthening teachers’ design competence can help prevent gamification from being used merely as a short-term novelty, thereby increasing its potential to generate lasting educational value.

This study highlights that gamified learning's value isn't in the technology but in supporting psychological needs, fostering motivation, and encouraging long-term learning. Combining psychological theory, instructional design, and technological application is key to turning innovation into sustained educational impact.

## Research limitations

Although this study achieved meaningful results, several limitations should be acknowledged. First, this study adopted a one-group post-intervention survey design rather than a randomized controlled experimental design. The study did not include a control group, a pretest–posttest comparison, or a comparison between gamified and non-gamified learning conditions. In addition, all data were collected through self-report questionnaires after the 16-week course. Therefore, the findings should be interpreted as evidence of associations among perceived gamification design elements, intrinsic motivation, and learning persistence, rather than as definitive causal evidence. Future studies should adopt randomized controlled trials, quasi-experimental designs with comparison groups, or longitudinal pretest–posttest designs to more rigorously examine the causal effects of gamified instructional design.

Second, the sample was drawn from a single university in Taichung, Taiwan. This limits the generalizability of the findings across different regions, institutions, disciplines, and educational systems. Future studies are needed to examine whether the results are transferable to different institutional and cultural contexts.

Third, this study relied on questionnaire data, which were primarily based on students’ self-reports. This may lead to social desirability bias or subjective perception bias. Future research could combine survey data with learning analytics data, classroom observations, interviews, or platform log data to provide a more comprehensive understanding of students’ motivation and learning persistence.

## Supplementary Information

Supplementary Material 1. Appendix 1. Questionnaire of gamification design elements. Appendix 2. Questionnaire of intrinsic motivation. Appendix 3. Questionnaire of learning persistence

## Data Availability

The original contributions presented in the study are included in the article/Supplementary Material; further inquiries can be directed to the corresponding author.
