# Supplementary material for: Gamification is not just fun: the effect of gamification design elements on shaping intrinsic motivation and learning persistence
Source: BMC Psychol. 2026 Jul 2;14:1346. doi: 10.1186/s40359-026-05046-8 (PMC13579831; doi:10.1186/s40359-026-05046-8)
Supplement: Supplementary file 1 [file 40359_2026_5046_MOESM1_ESM.docx]

**Appendix**

**1. Questionnaire of Gamification Design Elements**

| Dimension | Item |
| --- | --- |
| Autonomy Support | 1. I can choose how to participate in the learning activities provided by this system. |
|  | 2. When using this system for learning, I feel that I have control over my actions. |
|  | 3. I can complete the learning tasks offered by the system in my own way. |
|  | 4. This system allows me to learn in ways that align with my personal preferences. |
| Competence Support | 1. I feel capable of completing the learning tasks in this system.;;; |
|  | 2. The feedback provided by the system helps me understand my performance. |
|  | 3. When engaging in these learning activities, I feel confident in my performance. |
|  | 4. The gamification design elements clearly display my learning progress and growth. |
| Relatedness Support | 1. When using this system for learning, I feel connected to other learners. |
|  | 2. Participating in activities within the system with others gives me a sense of belonging. |
|  | 3. Team tasks or social challenges make me feel that I am part of a group. |
|  | 4. During the learning process, I can feel support from others. |

**2. Questionnaire of Intrinsic Motivation**

| Dimension | Item |
| --- | --- |
| Enjoyment | 1. I find participating in this online learning activity enjoyable.;; |
|  | 2. When engaging in this online learning activity, I experience a sense of enjoyment. |
|  | 3. I enjoy the process of participating in this online learning activity. |
| Curiosity | 1. The content of this online learning activity stimulates my curiosity. |
|  | 2. I am interested in the content presented in this online learning activity. |
|  | 3. Participating in this learning activity makes me want to learn more about related knowledge. |
| Challenge | 1. This online learning activity presents an appropriate level of challenge for me. |
|  | 2. The challenges in the learning activity motivate me to continue participating. |
|  | 3. Even when the learning content is difficult, I am willing to invest time to complete it. |

**3. Questionnaire of learning awareness**

| Dimension | Item |
| --- | --- |
| Behavioral Persistence | 1. When learning activities are challenging, I am still willing to persist in learning. |
|  | 2. Even when the course becomes difficult, I choose to continue participating. |
|  | 3. I am determined to complete all online learning tasks. |
|  | 4. When encountering problems, I do not give up easily, demonstrating a high level of persistence. |
| Sustained Engagement | 1. Throughout the entire course, I consistently participated in learning tasks. |
|  | 2. As time goes by, I am able to maintain my level of engagement in learning activities. |
|  | 3. Even when there are distractions, I can remain engaged in learning. |
|  | 4. Throughout the course, I continuously invest effort in learning activities. |
